# Gene trap mutation of murine Outer dense fiber protein-2 gene can result in sperm tail abnormalities in mice with high percentage chimaerism

**DOI:** 10.1186/1471-213X-10-67

**Published:** 2010-06-15

**Authors:** Heide Tarnasky, Min Cheng, Young Ou, Jacob C Thundathil, Richard Oko, Frans A van der Hoorn

**Affiliations:** 1 Department of Biochemistry & Molecular Biology, University of Calgary, Calgary, Canada; 2 Department of Production Animal Health, University of Calgary, Calgary, Canada; 3 Department of Anatomy & Cell Biology, Queen's University, Kingston, Canada

## Abstract

**Background:**

Outer dense fiber protein 2, Odf2, is a major component of the outer dense fibers, ODF, in the flagellum of spermatozoa. ODF are associated with microtubule doublets that form the axoneme. We recently demonstrated that tyrosine phosphorylation of Odf2 is important for sperm motility. In the course of a study of Odf2 using Odf2 mouse knockout lines we observed that males of a high percentage chimaerism, made using XL169 embryonic stem cells, were infertile, whereas mice of low-medium percentage chimaerism were fertile.

**Results:**

XL169 ES cells have a β-geo gene trap cassette inserted in the Odf2 gene. To determine possible underlying mechanisms resulting in infertility we analyzed epididymal sperm and observed that >50% displayed bent tails. We next performed ultrastructural analyses on testis of high percentage XL169 chimaeric mice. This analysis showed that high percentage XL169 chimaeric mice produce elongating spermatids that miss one or more entire outer dense fibers in their midpiece and principal piece. In addition, we observed elongating spermatids that show thinning of outer dense fibers. No other obvious abnormalities or defects are present in elongating spermatids. Spermatozoa from the caput and cauda epididymis of XL169 mice of high percentage chimaerism show additional tail defects, including absence of one or more axonemal microtubule doublets and bent tails. Sperm with bent tails display abnormal motility.

**Conclusions:**

Our results document the possible impact of loss of one Odf2 allele on sperm tail structure and function, resulting in a novel sperm tail phenotype.

## Background

The mammalian sperm tail contains several unique structures of poorly defined function. One of these structures, the outer dense fibers (ODF), is present in the sperm tail midpiece -together with the mitochondrial sheath- as well as the principal piece. Several major ODF proteins, including Odf1 and Odf2, have now been cloned by us and others [1-6], as have proteins that associate with these major ODF proteins. Our earlier work indicated that many ODF proteins and ODF-associated proteins interact using specific leucine zipper motifs [2,7,8]. Following on these studies it was discovered that some, but not all, ODF- and ODF-associated proteins, including Odf2 [9,10], are expressed in spermatids as well as in somatic cells.

Odf2 was discovered in our lab by virtue of its functional interaction with Odf1 [2]. Odf2 is a major ODF protein that can also self-interact. It has two leucine zipper motifs [2,6], one of which binds Odf1. The interacting proteins, if any, to the second leucine zipper are unknown. We recently found that Odf2 can bind cdk5, which upon activation can phosphorylate Odf1 [11]. ODF proteins have now also been implicated in sperm motility. We observed recently that inhibition of tyrosine phosphorylation of Odf2 and Tektin-2 adversely impacts sperm motility [12].

Although it was initially believed that Odf2 is testis-specific, it was discovered that differential splicing produces various forms of Odf2, one of which, Odf2/Cenexin1, is expressed in somatic cells [13-17] where it localizes to the centriole, in particular to the distal appendages on the mother centriole [9,18]. Odf2/Cenexin1 differs from Odf2 in that it contains a centriolar localization motif [15] and a different C-terminus that can bind polo-like kinase 1 (Plk1) required for bipolar spindle formation and regulation of mitotic progression [13,19]. Odf2/Cenexin1 is also important for formation of primary cilia [20]. These results suggested that the Odf2 gene may have different functions in somatic and male germ cells, which express different Odf2 protein variants.

Clues as to possible functions of Odf2 in mice were recently published [21]. The study described chimaeras generated using a gene trap RO072 embryonic stem (ES) cell line, which has an interruption of one of the Odf2 alleles. Chimaeras could be bred to generate heterozygote mice, but not homozygotes. The study concluded that one copy of Odf2 sustains normal development and male fertility, but a homozygous interruption of the Odf2 gene is incompatible with early development. No other mouse knockout models have studied the role of individual ODF components [22].

In the course of our investigation of Odf2 and its expression and function in male germ cells we had used a different Odf2^+/- ^ES cell line, XL169, and we observed that in contrast to our RO072 Odf2^+/- ^chimaeric results, high percentage XL169 chimaeric males were all infertile. XL169 ES cells have an interruption of one of the two Odf2 alleles at a different location -more towards the 5' end- than RO072 ES cells. Investigation of the possible basis for their infertility showed that they produce elongating spermatids and spermatozoa many of which displayed an abnormal phenotype, lacking one or more entire outer dense fiber. Epididymal spermatozoa presented with an accumulation of additional tail defects involving ODF and the axoneme and displayed abnormal motility.

## Results

### Generation of XL169-derived Odf2 chimaeras

**Figure 1:**
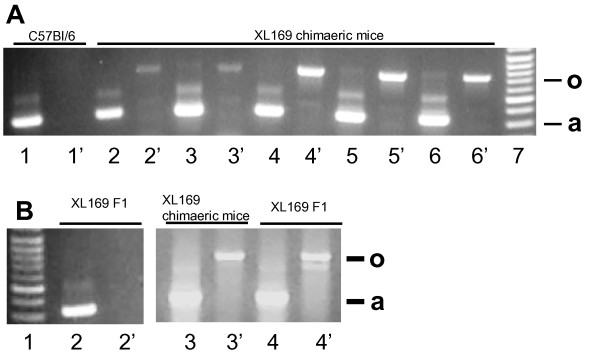
**Genomic analysis XL169 chimaeric mice and offspring**. A) DNA was extracted from tail snips of XL169 chimaeric mice and C57BL/6 control mice and analyzed by PCR for the insertion of the β-geo cassette between Odf2 exons 4 and 5. Each DNA sample was analyzed by primers specific for actin (lanes 1, 2, 3, 4, 5 and 6: "a") and primers specific for Odf2-β-geo (lanes 1', 2', 3', 4', 5' and 6'; "o"). Lane 7 shows DNA markers. The source of DNA was as follows: lanes 1 and 1', C57BL/6 (which does not have Odf2-β-geo DNA); lanes 3 - 6', XL169-derived chimaeric male mice with various percentage chimaerism. B) PCR was used to detect Odf2-β-geo DNA in testicular DNA of F1 offspring resulting from matings of XL169 chimaeric males with normal females. Each DNA sample was analyzed by primers specific for actin (lanes 2, 3 and 4; "a") and primers specific for Odf2-β-geo (lanes 2', 3' and 4'; "o"). Lane 1 shows DNA markers. The source of DNA was: lanes 2 and 3, agouti F1, which did not inherit Odf2-β-geo DNA; lanes 4 and 5, chimaeric male; lanes 6 and 7, agouti Odf2^+/- ^offspring.

XL169-derived chimaeric mice were made using the gene trap embryonic stem cell line XL169 (BayGenomics): the β-geo cassette used to interrupt one Odf2 allele is integrated between exons 4 and 5 (Additional file 1). The resulting interrupted allele has the potential to encode an Odf2-geo fusion protein, which however lacks the critical Odf2 leucine zipper that mediates binding to Odf1 (Additional file 1). Chimaeras were generated by injection of XL169 ES cells into C57BL/6 blastocysts. The presence of the β-geo cassette in chimaeras was determined by genomic PCR using DNA isolated from tail snips and confirmed by sequence analysis. In XL169 chimaeras an intronic PCR primer and the β-geo gene primer generated a specific 650 bp product with the exact expected sequence (Fig. 1A, lanes 2', 3', 4', 5', 6'). These results were confirmed using a different set of primers (Experimental procedures). Control C57BL/6 mice do not contain the β-geo interrupted Odf2 gene as expected (Fig. 1A, lane 1'). An actin control for DNA amount and quality was done by PCR for all samples (Fig. 1A, lanes 1, 2, 3, 4, 5, 6). We also generated chimaeric mice derived from the RO072 ES cell, in which the β-geo cassette is integrated in exon 9 [21], for a comparison of results (Additional file 1).

### Breeding results for Odf2 chimaeras

Chimaeras were mated to C57Bl/6 females. ES-derived offspring is expected to be agouti. We observed that all high percentage XL169 chimaeric males (>90 percent chimaeric) were infertile, producing neither black nor agouti offspring (Table 1). XL169 chimaeric mice of low-medium percentage chimaerism did produce agouti and black offspring (Table 1) indicating that XL169 ES cells can contribute to the germ line. Genotyping by PCR showed that the vast majority (156 of 162 animals) of agouti offspring of low to medium percentage XL169 chimaeric males are wt in Odf2, with only 6 confirmed Odf2^+/- ^male offspring (Fig. 1B). This suggested that spermatids that inherit the interrupted Odf2 allele do not have the same functionality as spermatids that inherit the wt Odf2 allele from XL169 cells. Four of six XL169-derived Odf2^+/- ^males were infertile, and two produced very small litters (1-2 offspring/litter). In comparison and in contrast to the results for XL169 chimaeric male mice, all RO072-derived chimaeric male mice were fertile, including all high percentage chimaeric males (Table 1).

**Table 1:** XL169- and RO072- derived chimaeric male Breeding Results

male XL169 mice analyzed^a^	total # offspring produced by males analyzed	agouti offspring produced^b^	heterozygotes produced
low-medium percentage chimaeric males^c^	494	162	6

high percentage chimaeric males^d^	0	N/A^e^	N/A

			

**male RO072 mice analyzed**	**total # offspring produced by males analyzed**	**agouti offspring produced**	**heterozygotes produced**

low-medium percentage chimaeric males^f^	301	46	8

high percentage chimaeric males^g^	75	11	2

Notes:

a) chimaerism was determined by coat color (agouti): over 90% chimaeric is classified as "high", below 90% as "low-medium".

b) agouti offspring can have the Odf2^+/+ ^genotype (phenotypically wt mice) or the Odf2^+/- ^genotype (heterozygote). In agouti offspring one Odf2 allele derives from the C57Bl/6 background and the other allele (either the normal Odf2 allele, or the genetrap-interrupted Odf2 allele) derives from the XL169 ES background

c) 13 males of low-medium % chimaerism were analyzed by breeding

d) 5 males of high % chimaerism were analyzed by breeding

e) N/A = not applicable.

f) 7 males of low-medium % chimaerism were analyzed by breeding

g) 2 males of high % chimaerism were analyzed by breeding

### RNA analysis of XL169 chimaeras

**Figure 2:**
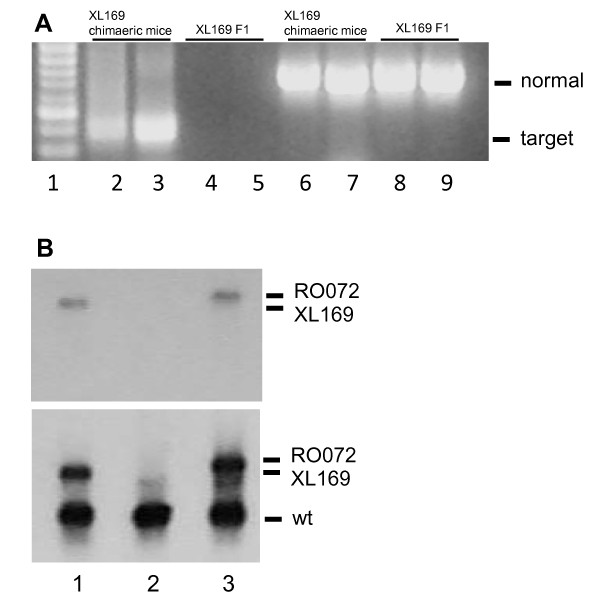
**Odf2-β-geo RNA is expressed in XL169 chimaeric mice**. A) We analyzed Odf2-β-geo RNA expression in RNA isolated from primary fibroblasts XL169 chimaeric ear clip cultures using RT PCR. Primers were specific for Odf2-β-geo (lanes 2 - 5; "target") or for wild type Odf2 (lanes 6 - 9; "normal"). Lane 1, DNA markers; lanes 2 , 3, 6 and 7, chimaeric males; lanes 4, 5, 8 and 9, Odf2^+/- ^F1 offspring . B) Northern blotting analysis was used to compare expression levels of wild type Odf2 RNA and Odf2-β-geo RNA in testis from XL169 and RO072 chimaeric mice. Top panel: blots were analyzed using a geo-specific probe that only detects Odf2-β-geo RNA ("RO072" and "XL169"). Bottom panel: RNA blots were analyzed using an Odf2-specific probe, which detects both Odf2 RNA ("wt") and Odf2-β-geo fusion RNA ("RO072" and "XL169"). The source of RNA was: lane 1, XL169 chimaeric male; lane 2, C57Bl/6 male; lane 3, RO072 chimaeric male.

The insertion of the cassette in XL169 cells predicts the expression of a fusion mRNA linking part of Odf2 to β-geo. We observed in preliminary RT PCR analysis that the targeted Odf2 allele (Odf2-β-geo) is expressed in XL169 ES cells. We used this approach to analyze expression of Odf2-β-geo RNA in XL169 chimaeric mice and their offspring. To do this, we established primary fibroblasts cultures from ear clips. RT-PCR analysis of RNA using exon 4 and β-geo-specific primers generated a 450 bp DNA product (Fig. 2A, lanes 2 and 3) indicative of expression of the targeted allele in XL169 chimaeric male mice. Sequence analysis of this PCR product confirmed that it represented the expected sequence of exon 4 region linked to the β-geo cassette sequence. Wild type Odf2 RNA, detected using Odf2-specific primers, is also expressed in these mice, as expected (Fig. 2A, lanes 6 and 7). In contrast to chimaeric mice, XL169 agouti Odf2^+/- ^offspring did not express Odf2-β-geo RNA (Fig. 2A, lanes 4 and 5), but -as expected- do express wild type Odf2 RNA (Fig. 2A, lanes 8 and 9). This suggests that the targeted Odf2 allele is silenced in Odf2^+/- ^male mice.

To confirm these results, we performed northern blot analysis of testis mRNA from XL169 and RO072 chimaeric males. The results show that XL169 chimaeric males express both an ~ 2.8 kb Odf2-β-geo mRNA and wild type 2.2 kb Odf2 mRNA in testis (Fig. 2B, lane 1), as expected. Similarly, RO072 chimaeric mice express wild type Odf2 mRNA and an ~ 3.2 kb RO072 Odf2-β-geo mRNA, which is larger due to a greater portion of Odf2 present in the fusion RNA (Fig. 2B, lane 3). Wild type mice did not show expression of Odf2-β-geo mRNA as expected (Fig. 2B, lane 2).

### XL169-derived chimaeric males produce sperm with morphological abnormalities

**Figure 3:**
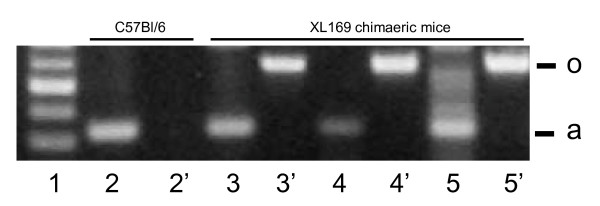
**Ejaculates from XL169 chimaeric males contain spermatozoa that carry Odf2-β-geo**. We analyzed whether or not high percentage chimaeric males produce spermatozoa and if they do whether or not these carry Odf2-β-geo DNA. Sperm ejaculates were indeed obtained from uterine horns of females mated to high percentage XL169 chimaeric mice and analyzed by genomic PCR using primers specific for Odf2-β-geo as described in Fig. 1 ("o"; lanes 2', 3', 4' and 5') and actin ("a"; lanes 2, 3, 4 and 5). Lane 1, marker DNA; lanes 2 and 3, C57Bl/6 sperm, which does not have Odf2-β-geo DNA; lanes 4 - 9, sperm from different chimaeric mice: note that they carry the Odf2-β-geo DNA.

The failure of high percentage XL169 chimaeric male mice to produce offspring could be due to several factors including an absence of spermatozoa. To investigate this possibility without sacrificing the high percentage XL169 chimaeric mice we first analyzed if we could collect sperm ejaculates from uterine horns of mated females. This was successful demonstrating that these mice do produce sperm. We also analyzed these ejaculates for the presence of Odf2-β-geo DNA by PCR. We also analyzed sperm ejaculates produced by low-medium percentage XL169 chimaeric males. The results indicate that Odf2-β-geo DNA is detectable in sperm ejaculates of all XL169 chimaeric mice, including high percentage XL169 chimaeric mice (Fig. 3, lanes 5, 7 and 9). Sperm from wild type mice do not carry Odf2-β-geo DNA, as expected (Fig. 3, lane 3). All samples were analyzed for the amount and quality of DNA using actin primers, as control (Fig. 3, lanes 2, 4, 6 and 8). These data show that high percentage XL169 mice can produce sperm and that some must be carrying the targeted Odf2 allele.

**Figure 4:**
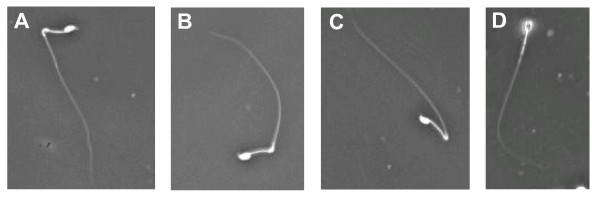
**Abnormal sperm produced by high percentage XL169 chimaeric male mice**. Spermatozoa were isolated from cauda epididymis of high percentage XL169 chimaeric males and analyzed by light microscopy (computer assisted sperm analysis equipment). Over 50% of all sperm displayed bent tails. Panels A - C: examples of sperm with bent tails. Panel D: example of sperm with an apparent normal morphology. Magnification, 40×.

In the course of these experiments we noticed that ejaculates of high percentage XL169 chimaeric mice contained many sperm (>50%) with bent tails. To confirm this, we isolated epididymal sperm from high percentage XL169 chimaeric mice and observed that >50 percent has abnormal tail morphologies, many displaying bent tails (Fig. 4, panels A - C). Sperm from normal mice and low-medium percentage XL169 chimaeric animals only showed a small percentage (<5%) of abnormal sperm. The remainder of spermatozoa had an apparently normal morphology (Fig. 4, panel D). We used computer assisted sperm analysis equipment and software to record the motility of sperm with bent tails. We observed that sperm from high percentage XL169 chimaeric males display abnormal motility (Additional file 2 shows a movie of examples of sperm with bent tails and abnormal motility).

**Figure 5:**
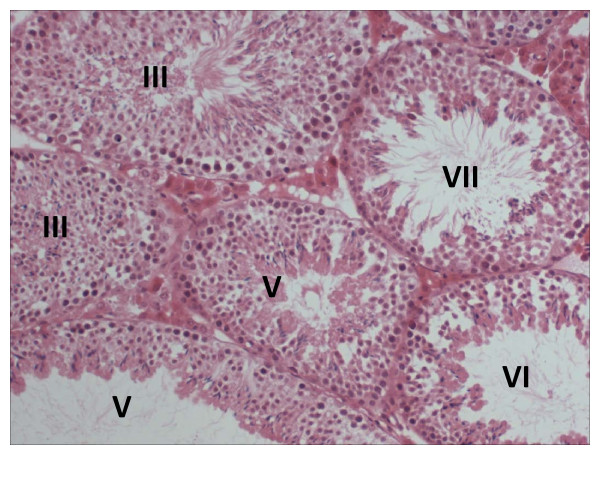
**Histology of testis sections of high percentage chimaeric XL169 mice**. Testis sections of high percentage XL169 chimaeric animals were prepared from Bouin's fixed tissue, stained with haematoxylin and eosin, and analyzed by light microscopy. The stages of the cycle of the seminiferous tubules are indicated in Roman numerals. Magnification 10×.

**Figure 6:**
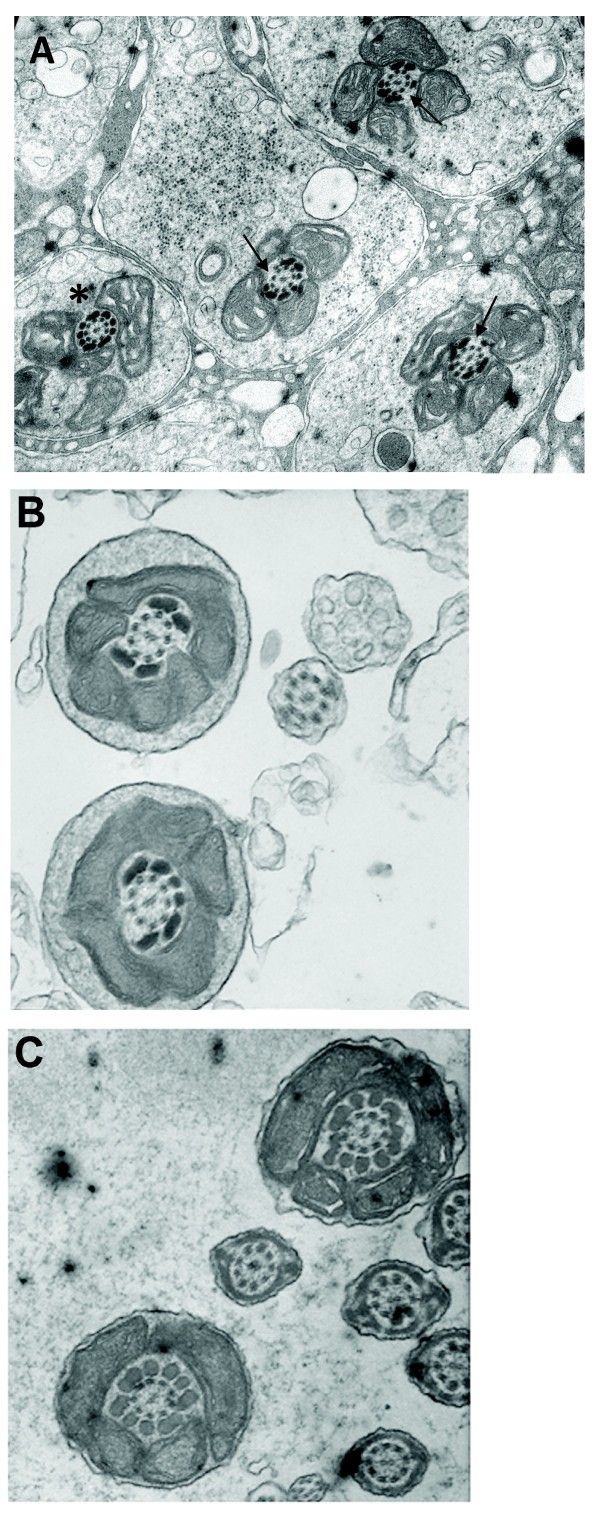
**Abnormal ODF morphology in elongated spermatids of high percentage XL169 chimaeric males**. To study the possible basis for the infertility of high percentage XL169 chimaeric mice, we carried out an ultrastructural electron microscopic analysis of samples from testis in a comparison to testis from low-medium percentage XL169 chimaeric mouse. Panel A shows an example of connected step 14-15 spermatids belonging to one syncytium, some of which appear normal (star), others lack ODF (arrows). Panel B shows two examples of abnormal midpieces from step 16 spermatids, one of which misses five ODF. Panel C shows step 16 spermatid midpieces from a low-medium percentage XL169 chimaeric mouse that appear normal. Magnification is 28,000 ×.

**Figure 7:**
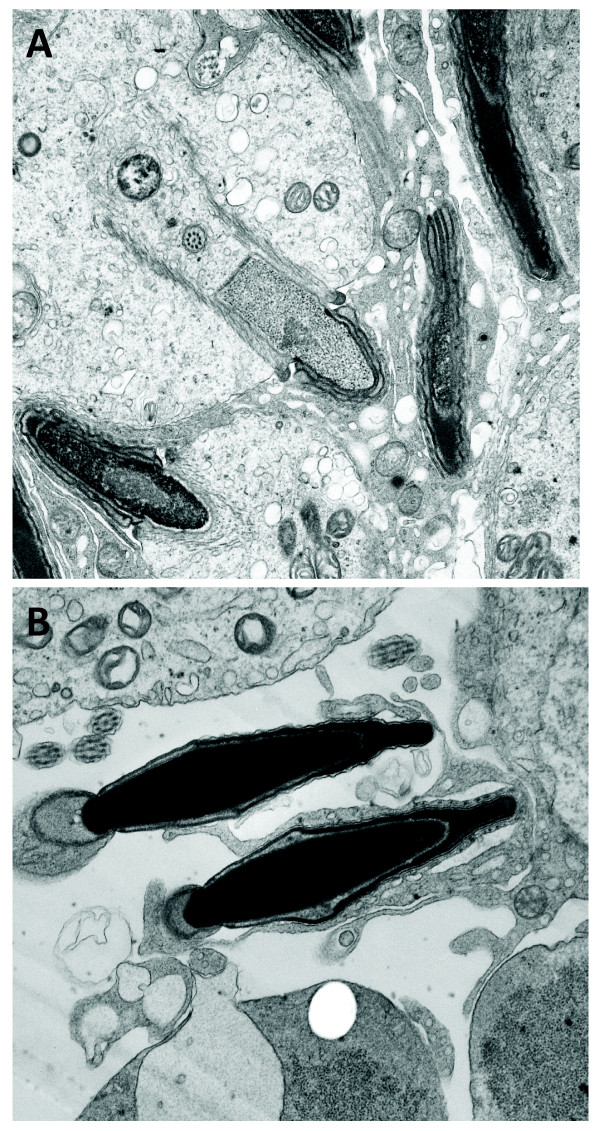
**Spermatids and spermatozoa from high percentage XL169 chimaeric mice display normal heads**. Ultrastructural analysis of developing spermatid heads from high percentage XL169 chimaeric males was carried out. Panel A shows examples of the normal development of heads of step 10 spermatids. Panel B shows two examples of normal heads of step 16 elongated spermatids (stage VIII) about to be released from Sertoli cells during spermiation. Magnification is 28,000 ×.

To study the basis for this observation we carried out ultrastructural analysis of spermatids and epididymal samples in a comparison with samples from normal mice and low-medium percentage chimaeric XL169 mice. First, testis sections of high percentage XL169 chimaeric mice show an apparently normal histology (Fig. 5). Sperm counts from high percentage XL169 chimaeric mice and control mice were also comparable. Next, the results of ultrastructural analyses of high percentage XL169 chimaeric testis show that over half of the spermatids in high percentage chimaeric XL169 males exhibit ODF defects. Fig. 6A shows an example of a group of step 14-15 spermatids derived from the same clone connected by intracellular bridges where one midpiece appears normal (star) flanked by spermatid midpieces that lack ODF to varying degrees (arrows). The axoneme is normal. We noticed instances of spermatid midpieces that lacked up to 5 entire ODF (Fig. 6B: arrow). It is possible that spermatids without a gross major defect have changes that escaped detection by this method. Spermatids from wild type mice (not shown) and from low-medium percentage XL169 chimaeric mice (Fig. 6C) did not show the absence of entire ODF. No other defects were observed in spermatids from high percentage XL169 chimaeric mice: Fig. 7A shows that step 10 spermatids of high percentage XL169 chimaeric mice have a developing sperm head exhibiting a normal morphology. Similarly, at spermiation (stage VIII) the morphology of spermatid heads in step 16 is normal (Fig. 7B). We did not observe any other clear abnormalities other than missing ODF in spermatids.

**Figure 8:**
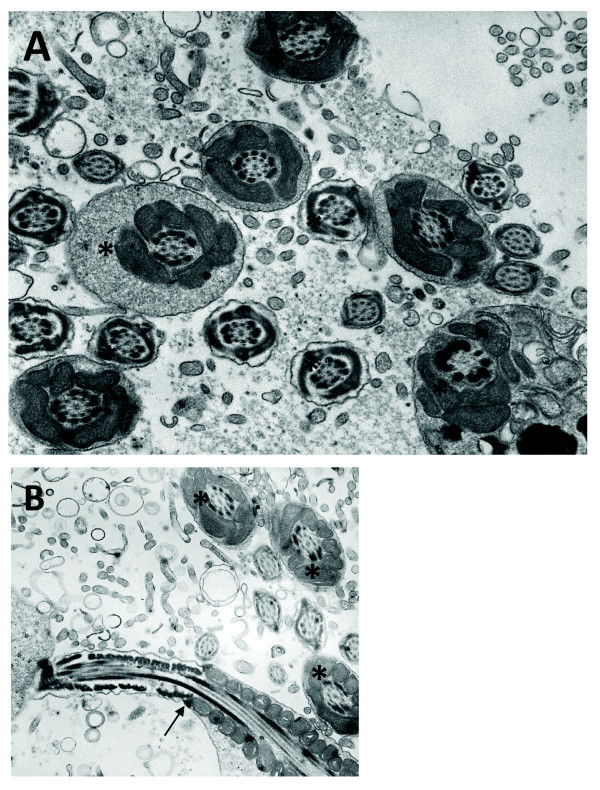
**Caput epididymal spermatozoa of high percentage XL169 chimaeric males**. Analysis of the sperm tails of high percentage XL169 chimaeric males in the caput indicate that sperm tails lack ODF (indicated by stars) but do not display additional gross abnormalities. Panel A shows midpieces and principal pieces of caput spermatozoa. Panel B shows several midpieces lacking various numbers of ODF, as well as a tail showing mild bending. The annulus of the tail (arrow) appears normal. Magnification, 28,000×.

We next examined the caput epididymis of high percentage XL169 chimaeric mice and found that -as expected from the results for elongating spermatids- spermatozoa miss one or more ODF from the midpiece and principal piece (Fig. 8A and 8B). We did not observe significant bending of sperm tails in samples from caput epididymis, indicating that the bending of tails observed in ejaculates occurs later. Fig. 8B documents that the annulus (arrow) of caput sperm has a normal morphology.

**Figure 9:**
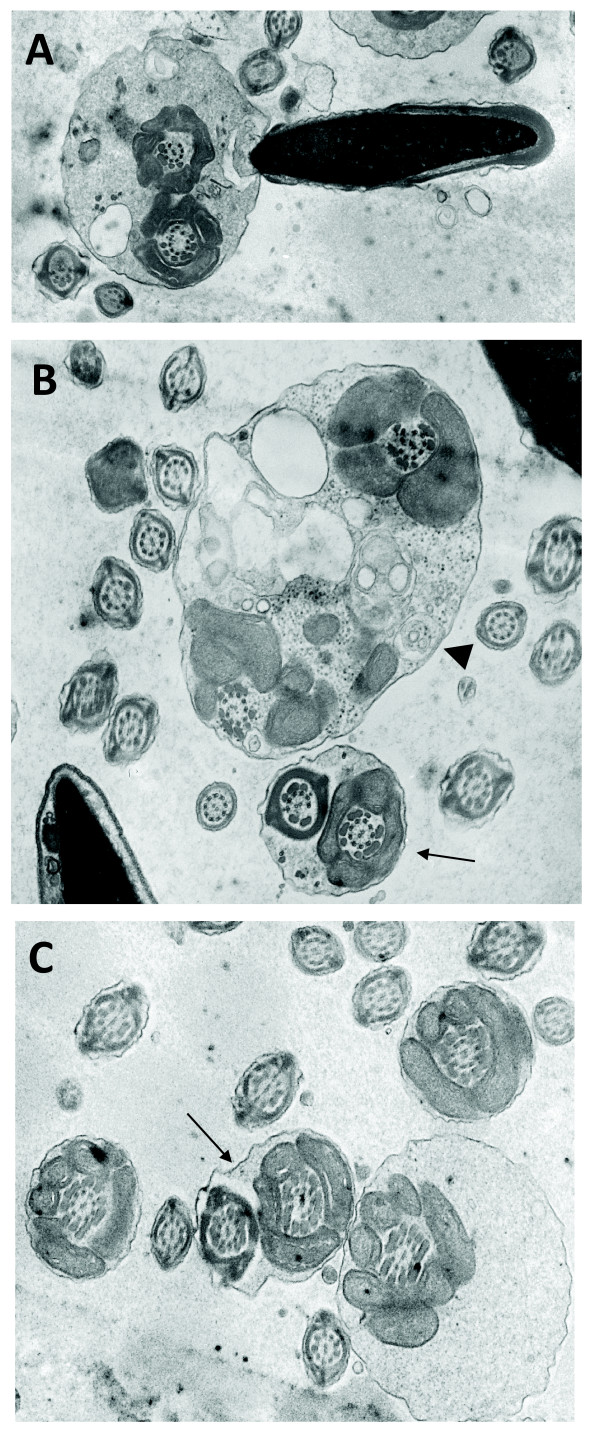
**Cauda epididymal spermatozoa of high percentage XL169 chimaeric males acquire additional defects**. In cauda epididymis we noted the presence of many spermatozoa with the bent tails. Panel A shows a spermatozoon with a normal head and a bent tail resulting from the midpieces folding and fusing on itself. Note the absence of several ODF. Panel B shows that the bending can result in fusion of the midpiece (arrowhead) (seen in the majority of cases), as well as fusion of the midpiece and principal piece (arrow). Panel C illustrates that on occasion a fusion as a result of bent tails was observed in spermatozoa from low-medium percentage XL169 chimaeric animals. Magnification is 45,000 ×.

Interestingly however, when analyzing cauda epididymis of high percentage XL169 chimaeric mice we observed many bent tails as well as defects in the axoneme, with one or more axonemal microtubule doublets missing. First, as shown in Fig. 9A (and more examples in Additional file 3) we noted a majority of spermatozoa with a fusion of the midpiece region demonstrating that bending of the tail must have occurred above the annulus. Also, as expected these midpieces (and principal pieces) also miss a variable number of ODF and/axonemal microtubule doublets. Head morphology appears normal. Fig. 9B shows a spermatozoon with a bent tail fused midpiece region close to another spermatozoon with a fusion of the midpiece and principal piece regions. Additional file 3 shows more examples of bent tails, each with loss of ODF, resulting in fusion of head and tail (panel A) and showing the curve of a bent tail (panel B). It is important to note here that we never observed these additional defects, including bent tails, in spermatids within the testis of high percentage XL169 chimaeric mice, which only display loss of ODF. Only rarely did we observe fusion of bent tails in spermatozoa from cauda epididymis of low-medium XL169 chimaeric mice (Fig. 9C: arrow) and tails did not appear to miss entire ODF in midpiece or principal piece. We have not observed bent tails in spermatozoa from wild type mice.

## Discussion

Here we report that XL169 Odf2^+/- ^chimaeric male mice with a high percentage chimaerism are infertile and display a novel abnormal sperm tail phenotype. Elongated spermatids exhibit the absence of one or more ODF from the midpiece and principal piece, but have normal head development and annulus. In spermatozoa from cauda epididymis we noted the presence of additional tail defects, including loss of axonemal microtubule doublets and bent tails.

### An abnormal ODF sperm tail phenotype

Our ultrastructural analysis of male germ cells from high percentage XL169 chimaeric mice identified elongated spermatids that displayed reduced numbers of ODF in the sperm midpiece and principal piece. The head, acrosome and annulus of spermatids of high percentage XL169 males appeared to have a normal morphology. No other defects have been identified by us in such spermatids. Since the only known mutation in the XL169 animals is the targeting of one Odf2 allele, it is reasonable to propose that this mutation is linked to the observed ODF abnormalities. We also observed the appearance of additional tail abnormalities in cauda epididymal sperm: axonemal abnormalities seen in cauda spermatozoa and bent tails. These additional defects are not present in elongated spermatids, and indeed caput spermatozoa only display a very mild bending if at all. This suggests that the additional defects are acquired during epididymal transit rather than originate while elongated spermatids still reside in the testis. The acquisition of additional sperm tail defects upon passage through the epididymis had been described previously in bovine and was shown to be related to the acquisition of the capacity for motility during epididymal transit [23]. Those results indicated how primary defects of the tail acquired in the testis can predispose the sperm to secondary defects acquired in transit through caput and cauda [23], a result we now document here in the mouse. This suggests that for normal sperm tail development the presence of a normal complement of ODF is essential: the lack of a normal complement of ODF may compromise the ability of the sperm tail to withstand forces associated with passage through the epididymis where they acquire the capacity for motility causing further damage. Normally, ODF are associated with each of 9 microtubule doublets in the sperm tail midpiece and with 7 of 9 microtubule doublets in the principal piece. The remaining two microtubule doublets are associated with the columns of the fibrous sheath (FS), which replace ODF numbers 3 and 8. Thus, the absence of ODF appears to contribute to further axonemal defects.

The molecular basis for the observed ODF defects may be associated with a reduced amount of Odf2 protein available for ODF morphogenesis as a result of interruption of one of the Odf2 alleles by the gene trap. It had been shown previously that spermatids form syncytia of cells connected by intercellular bridges, which allow the transfer of proteins including protamine [24] and transition proteins [25]. Thus, Odf2 protein, synthesized in an XL169-derived spermatid carrying the wild type Odf2 allele, may be redistributed to connected spermatids carrying the interrupted Odf2 allele. The degree of this redistribution is unknown. It is also not clear if synthesis of Odf2 protein from one allele suffices for production of ODF in two connected spermatids. However, redistribution must take place in principle since we did not observe spermatids lacking all ODF. We did observe that in groups of developing spermatids, all part of a syncytium, some appear normal while others miss one or more ODF. In contrast to the results for the XL169 line, high percentage RO072 chimaeric males are fertile. One important difference between these two lines is that the Odf2-β-geo fusion protein encoded by the gene trap-interrupted allele in RO072 animals is larger than and may have residual activity, compared to the much smaller fusion protein in XL169 animals (see Additional file 1). This possibility and a possible dominant negative role of the putative fusion protein in XL169-derived animals remain to be analyzed.

It is not clear why low to medium percentage chimaeric animals can produce agouti offspring. Both low-medium percentage and high percentage XL169 chimaeric males produce sperm, yet high percentage XL169 chimaeric males do not produce agouti offspring. In addition, the defects including bent tails observed in high percentage XL169 chimaeric spermatids are only rarely observed in spermatids of low-medium percentage XL169 chimaeric males. This suggests that additional, currently unknown, factors probably play a role in the observed infertility. One possible factor is that while in high percentage XL169 chimaeric animals practically all spermatids must be derived from the original XL169 ES cells, it is unknown what the percentage of such cells is in testis of low to medium percentage animals and we do not know the possible influences between these groups of spermatids. Another possibility is that in high percentage XL169 chimaeric animals practically all testicular supporting cells (including Sertoli cells and Leydig cells) only have one normal Odf2 allele, while low-medium percentage animals harbor supporting cells of wild type background. The effect on development and differentiation of supporting cells is not known. Finally, an observation that may contribute to the observed infertility is the inability to support early embryogenesis. We have observed (see Additional file 4) that matings of wild type female mice with high percentage XL169 chimaeric male mice result in abnormal embryo development (blocked embryogenesis, abnormal cell division; panels A - C) in comparison to normal embryos (panel D- 1.5 day embryo; panel E - 2.5 day embryo). This resembles previous reports showing that bovine sperm with morphological abnormalities fail during fertilization, or if fertilization does occur, resulting zygotes fail during early embryo development [26,27]. This failure may be the result of reduced amounts of Odf2.

ODF abnormalities have only been reported in few cases in samples either from infertile humans or mouse knockout experimental systems [22]. For example it was recently shown that selenium-deficiency in rats results in production of abnormal sperm that lack one or more axonemal microtubule doublets of the axoneme as well as the corresponding ODF [28]. This is in agreement with our earlier studies [7] that suggested that the axonemal doublet functions in part as anchor for the formation of the corresponding ODF: we demonstrated that Spag4 binds to the doublet followed by binding of Odf1 and other ODF proteins to Spag4. In a large mouse mutagenesis study [29] the Repro2 mutation was shown to result in elongated spermatids with an apparently normal axoneme, some aligned mitochondria and a disorganized array of ODF in the cytoplasm: these mice also suffer from dysplasia of the fibrous sheath [30]. Sperm tail abnormalities in humans have been classified in two groups [31-33]: a heterogeneous set of different sperm abnormalities as seen in low frequency in infertile men, and a specific set of sperm abnormalities seen in individuals. Abnormalities include axonemal irregularities, FS dysplasia [33-35] and sperm head abnormalities [31]. A recent case report of an infertile man with severe asthenozoospermia indicated an absence of axoneme and outer dense fibers in the principal piece [36]. It was not documented what the primary defect in spermatids was, but since the defects were observed in over 95% of sperm, it was concluded that a genetic defect may lie at the origin of the condition. It is possible, but unknown, if Odf2 mutations underlie this observed phenotype.

## Conclusion

The XL169 mouse high percentage chimaeric model adds to this gamut of sperm tail defects the abnormal ODF phenotype described here. The chimaeric mouse XL169 model provides a basis for further analysis of the contribution of ODF and Odf2 to sperm tail development during epididymal passage, to motility characteristics and competency to undergo capacitation. Our observations support a role for ODF and Odf2 in fertility.

## Methods

### Generation of recombinant Odf2 chimaeric mice

The gene trap embryonic stem cell lines XL169 and RO072 had been acquired from BayGenomics. The XL169 cells contain an insertion between exon 4 and exon 5 of the Odf2 gene of a promoter-less β-galactosidase neomycin fusion gene (β-geo): the insertion occurred at position 6312 in the Odf2 mouse genomic sequence. RO072 cells have the insertion of this cassette in exon 9 [21]. The insert contains a splice acceptor site, resulting in synthesis of an mRNA harboring Odf2 sequences linked to β-geo. To generate chimaeric mice XL169 and RO072 ES cells were injected into C57BL/6 mouse blastocysts at the Clara Christie Centre For Mouse Genomics at the University of Calgary. Chimaeric mice were obtained that showed varying degrees of chimaerism as determined by percentage agouti coat color and were maintained and bred at the Health Sciences Animal Resource Centre according to guidelines and regulations of the Canadian Council on Animal Care.

### Genotyping

Tail DNA: Genomic DNA was isolated from tails of chimaeric males and females as well as F1 offspring for PCR analysis as described before [37]. In short: tail snips were introduced into 600 μl of lysis buffer (100 mM NaCl, 20 mM Tris pH 7.5, 50 mM EDTA, pH8.0, 0.5% SDS and 5 μl Proteinase K at 20 mg/ml) and left at 55°C overnight in a rocking incubator. Tubes were spun at 10,000 rpm for 10 minutes and supernatant poured into clean tubes. One ml of 95% EtOH was added to precipitate DNA, which was collected with a glass rod and washed in 70% EtOH. The sample was air dried and resuspended in 80 μl of 10 mM Tris pH8.0. In indicated instances the Viagen Biotech Inc. Direct PCR Lysis Reagent (Tail) was used to isolate tail snip DNA for PCR analysis. Sperm DNA: To isolate DNA from ejaculated sperm, female C57BL/6 mice were mated with indicated Odf2^+/- ^chimaeric male mice and sperm was isolated between 0-2 hours post plug formation: the uterine horn was removed and gently squeezed to release sperm into 1× PBS solution. Isolated sperm ejaculates were frozen in PBS at -80°C.

Genomic PCR: The insertion site of the β-geo cassette into the Odf2 gene in XL169 ES cells was determined using the forward intronic primer F6201 (5' CTCATGTCCCTACTCAAGCTATAC 3') and β-geo primer 1 (5' AGTATCGGCCTCAGGAAGATCG 3'). PCR products were analyzed by electrophoresis on 1% agarose gels and a 650 bp DNA fragment was isolated using the QIA Quick gel extraction kit (Qiagen). Sequence analysis confirmed an insertion site of the β-geo gene between Odf2 exons 4 and 5 (Ensembl ENSMUSG00000026790; GenBank NM_013615.3).

Genotyping of chimaeric mice and F1 offspring was carried out using the Neomycin Primer 4761R (5' GAGAGGCTATTCGGCTATG 3') and Neomycin Primer 5493F (5' CAAGAAGGCGATAGAAGGC 3'). This primer set generates a product of approximately 700 bp in length in mice that are positive for the β-geo gene.

### RT-PCR analysis

Total RNA was extracted from mouse ear primary fibroblasts as follows: mouse ear clips from chimaeric and F1 mice were digested with 0.5 mg/ml collagenase and 0.01 μg trypsin in 500 μl DMEM medium supplemented with Sodium Pyruvate (Gibco) and penicillin/streptomycin (Sigma). Samples were digested for 30 min at 37°C. Using glass pipettes, ear clips were pipetted repeatedly to break up the tissue until full digestion. Samples were spun at 4000 rpm for 5 min at 4°C and washed 4 times in DMEM. Cells were pipetted into a 150 mm tissue culture plate and incubated in a humidified CO_2 _tissue culture chamber for one week. Upon reaching near confluence RNA was isolated from the primary fibroblast cultures using TRIZOL reagent (Invitrogen).

Total RNA was transcribed into cDNA using MMLV RT polymerase using oligo dT primers as described previously [38]. The subsequent PCR reaction was carried out using the cDNA and two different sets of primers. One set was specific for the junction between the Odf2 and β-geo sequences and the other set was specific for the wild type Odf2 allele. Primers used to confirm the presence of β-geo inserted into the Odf2 sequence were the New Exon (NE3) primer 3 (5' GGGACACCGTGAATGTACG 3') and the gene trap β-geo primer # 1 (5' AGTATCGGCCTCAGGAAGATCG 3'). A second set of primers used to analyze the presence of β-geo in F1 offspring were the NE3 primer and Primer #1C (5' CGAAGTTATCGCAGATCTGGACTCTAG 3'). Primers specific for the wild type Odf2 allele were primers NE3 and Exonic Primer 9 (5' CAGACGCTTCAGTAATGTACC 3'). PCR was done on a Perkin Elmer 2400 machine. Conditions were as follows: 95°C 4 min, followed by 45 cycles of (95°C for 30 sec, 55°C for 45 sec and 68°C for 1 min) , followed by 72°C 5 min and cooling to 4°C. PCR products were analyzed by agarose gel electrophoresis and sequencing of isolated fragments.

### Northern Blot analysis

Northern blot analysis of RNA was carried out essentially as described [2]. RNA samples used for northern blot analysis were prepared as follows: 4.7 μl RNA samples were mixed with 0.3 μl 1 M Na phosphate pH 6.5, 2.5 μl 37% formaldehyde, 7.5 μl formamide and DEPC-treated water and were heated to 65°C for 5 min. Samples were placed on ice and 5 μl of sample buffer containing 50% glycerol/50% formamide with Bromo-Phenol Blue and XCFF was added. The agarose gels contained 1.2% agarose, 6.2% formaldehyde and 20 mM Na-phosphate, pH6.5. Running buffer for the gel contained 6.5% formaldehyde and 20 mM Na-phosphate, pH 6.5. Gels were run at 90 mAmp for 2.5 hours and separated RNA was transferred to a Duralon membrane (Stratagene) overnight. The RNA on the membranes was linked using UV (Stratagene, Strata-linker) and analyzed with two probes. The lacZ probe is specific for β-galactosidase and was generated using the β-geo forward primer #3 (5' GGCGTTACCCAACTTAATCG3') and the β-geo reverse primer #4 (5' CGTCGGCGGTGTGCAGTTCAACCACC 3') and a lacZ containing plasmid. These primers generate a 902 bp lacZ probe. The second probe was specific for Odf2 and was generated using the Odf2 Exon 3 forward primer (5' GGGACACCGTGAATGTACG 3') and the Odf2 Exon 9 reverse primer (5' cagacgcttcagtaatgtacc 3') and an Odf2 cDNA. This primer set generates a 685 bp Odf2 probe.

Radiolabeled probes were made by PCR using ^32^P-dCTP: reactions contained 70 ng/μl of gel-isolated probes, ^32^P-dCTP, 1 mM cold dCTP and 1 μl 10 μM primers. PCR was carried out as follows: 2 min at 95°C; 35 cycles of (30 sec at 95°C, 1 min at 55°C and 1 min at 68°C); followed by 5 min at 72°C for and cooling to 4°C. Probes were purified using a Pharmacia Nick Column (Pharmacia) and boiled for 10 min at 95°C and cooled on ice for 5 min prior to addition to 30 mls of Na-Phosphate buffer (0.25 M Na-phosphate, pH 7.4, 1 mM EDTA, 7% SDS). Blots were left in a shaker overnight at 65°C and then removed and rinsed at RT in 2 × SSC/0.1% SDS for 5 min. Blots were next washed for 15 min at RT in 2 × SSC/0.1% SDS. Finally, blots were washed at 65°C in 0.1 × SSC/0.1% SDS for 30 min and rinsed in 0.1 × SSC at RT for 5 min prior to exposure to Kodak film.

### Ultrastructural analysis of testis sections and epididymal sperm

Testes and epididymides were collected from chimaeric mice. One forth of one testis was cut in 0.2 M cacodylate buffer, pH 7.2, containing 5% glutaraldehyde (fixative 1) to generate pieces of 1-2 mm^3 ^that were immersed in 1 ml of this fixative for 3 h at room temperature and stored at 4°C until post fixing in 1% osmium tetroxide and processing for epon embedding. Another quarter of the testis, destined for immune-electron microscopy, was cut as above but immersed in 100 mM phosphate buffer, pH 7.2, containing 4% paraformaldehyde and 0.8% glutaraldehyde (fixative 2) and then processed for LR White embedding. The remaining half of the testis was immersed in Bouin's fixative and then processed for paraffin embedding and routine histological evaluation. The caput and cauda compartments of one of a pair of epididymides were also processed as described above using fixative 1. The cauda of the other epididymis was nicked by razor blade several times in Na+K- phosphate buffer to allow sperm to flow out into the medium and be collected. The sperm were then centrifuged and processed as described above for fixative 2. Ultrathin sections from epon embedded tissue were mounted on copper grids and counterstained with uranyl acetate and lead citrate while ultrathin sections from LR White embedded tissue were mounted on formvar-coated nickel grids and used for immunocytochemical labeling before counterstaining and examination under a Hitachi 7000 transmission electron microscope.

## Authors' contributions

HT carried out all breeding and animal work, carried out PCR and wrote a first draft. MC provided support for PCR experiments. YO optimized PCR analysis of genomic DNA. JT provided computer assisted sperm analysis equipment and data. RO carried out the electron microscopy and ultrastructural analysis. FVDH conceived the project, obtained funding, supervised the entire project and wrote the manuscript. All authors read and approved the final manuscript.

## Supplementary Material

Additional file 1 **Schematic representation of the gene trap-interrupted Odf2 allele**. Shown is the location of the insertion sites of the geo gene trap cassette in the Odf2 allele in XL169 cells and in RO072 cells. The corresponding site of interruption in the Odf2 protein is also shown. The interrupted allele has the potential to encode fusion proteins between Odf2 and geo. Indicated are schematically the Odf2-geo fusion proteins expected in RO072 cells and XL169 cells. Click here for file

Additional file 2 **Sperm from high percentage XL169 chimaeric males exhibit abnormal motility**. Sperm from cauda epididymis of high percentage XL169 chimaeric mice were monitored using Computer Assisted Sperm Analysis equipment and software. Shown is a video (WMF format) of the movement of bent sperm. Click here for file

Additional file 3 **Ultrastructural analysis of spermatozoa of high percentage chimaeric XL169 mice**. Sections of cauda epididymis of high percentage XL169 chimaeric animals were analyzed by electron microscopy. Shown are examples of bent tails. Panel A shows a bent tail causing a fusion of the principal piece with the midpiece and the head. Panel B shows a section through a bent tail. Magnification, 28,000×. Click here for file

Additional file 4 **Abnormal early development of embryos produced by high percentage XL169 chimaeric male mice**. Embryos were collected at 1.5 days p.c. and 2.5 days p.c. from normal females mated to high percentage XL169 chimaeric males (panels A - C) or wild type males (Panels D, E). Embryos were immediately fixed after recovery and examined. Note the variation in size between cells and abnormal appearance within XL169-derived embryos compared to embryos (panel D - 1.5 days; panel E - 2.5 days) sired by wt mice. Magnification, 20×. Click here for file
