# Supplementary material for: Gene trap mutation of murine Outer dense fiber protein-2 gene can result in sperm tail abnormalities in mice with high percentage chimaerism
Source: BMC Dev Biol. 2010 Jun 15;10:67. doi: 10.1186/1471-213X-10-67 (PMC2894780; doi:10.1186/1471-213X-10-67)
Supplement: Additional file 1 — Schematic representation of the gene trap-interrupted Odf2 allele. Shown is the location of the insertion sites of the geo gene trap cassette in the Odf2 allele in XL169 cells and in RO072 cells. The corresponding site of interruption in the Odf2 protein is also shown. The interrupted allele has the potential to encode fusion proteins between Odf2 and geo. Indicated are schematically the Odf2-geo fusion proteins expected in RO072 cells and XL169 cells. [file 1471-213X-10-67-S1.DOC]

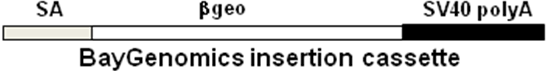

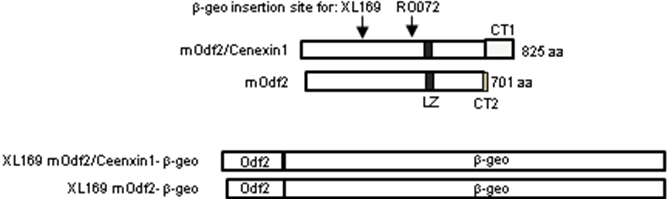

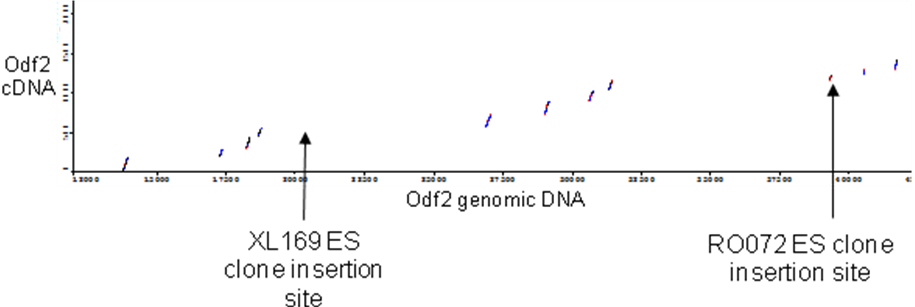


1. The gene trap cassette contains a promoter-less β-gal-neo (β-geo) cassette and an efficient splice acceptor site (SA). The insertion sites of the cassette in XL169 cells and in RO072 cells in the Odf2 gene were determined by genomic sequence analysis and are indicated.
2. The DNA insertion events can result in the production of Odf2-β-geo fusion proteins. The site of insertion of the β-geo coding region is indicated in the schematic of the Odf2 proteins. For XL169 the putative fusion protein retains 234 N-terminal residues of mOdf2 (“Odf2”), linked to β-geo as indicated, and for RO072 the putative fusion protein retains 424 N-terminal residues of mOdf2. Neither of the putative fusion proteins contain the leucine zipper (LZ) , which mediates interaction of Odf2 with ODF proteins, or the C-termini CT1 or CT2, important for function in the centrosome.

**A**

**B**
