# Supplementary figures and images for: Gene trap mutation of murine Outer dense fiber protein-2 gene can result in sperm tail abnormalities in mice with high percentage chimaerism
Source: BMC Dev Biol. 2010 Jun 15;10:67. doi: 10.1186/1471-213X-10-67 (PMC2894780; doi:10.1186/1471-213X-10-67)

## Slide 1
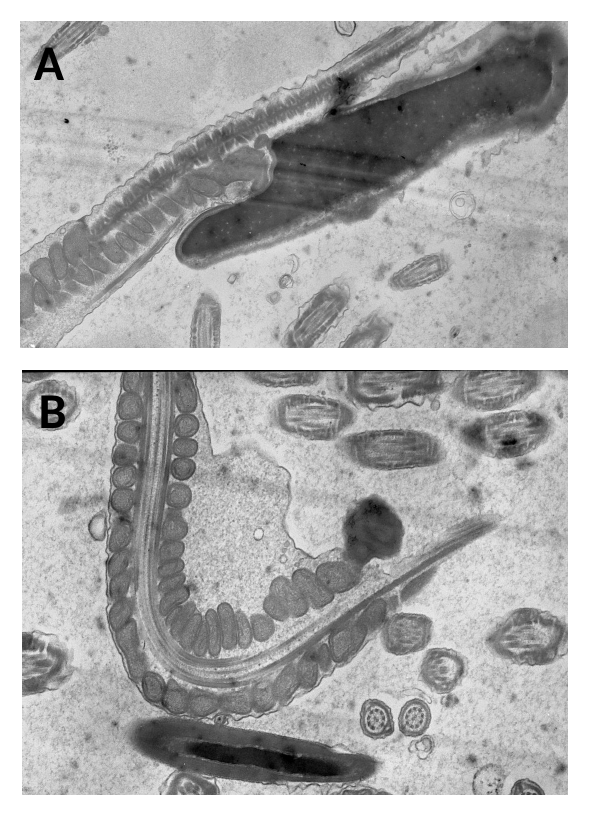

A
B

Supplement: Additional file 3 — Ultrastructural analysis of spermatozoa of high percentage chimaeric XL169 mice. Sections of cauda epididymis of high percentage XL169 chimaeric animals were analyzed by electron microscopy. Shown are examples of bent tails. Panel A shows a bent tail causing a fusion of the principal piece with the midpiece and the head. Panel B shows a section through a bent tail. Magnification, 28,000×. [file 1471-213X-10-67-S3.PPT]

## Slide 1
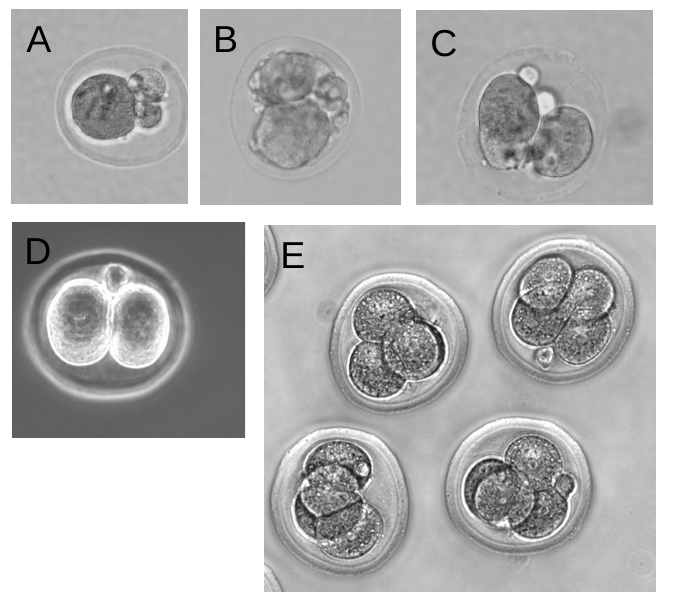

A
B
C
D
E

Supplement: Additional file 4 — Abnormal early development of embryos produced by high percentage XL169 chimaeric male mice. Embryos were collected at 1.5 days p.c. and 2.5 days p.c. from normal females mated to high percentage XL169 chimaeric males (panels A - C) or wild type males (Panels D, E). Embryos were immediately fixed after recovery and examined. Note the variation in size between cells and abnormal appearance within XL169-derived embryos compared to embryos (panel D - 1.5 days; panel E - 2.5 days) sired by wt mice. Magnification, 20×. [file 1471-213X-10-67-S4.PPT]
